# Prevalence of Metabolic Syndrome and Nonalcoholic Fatty Liver Disease among Premenopausal and Postmenopausal Women in Ho Municipality: A Cross-Sectional Study

**DOI:** 10.1155/2020/2168381

**Published:** 2020-04-29

**Authors:** Anita Mawuse Setroame, Patrick Kormla Affrim, Albert Abaka-Yawson, Precious Kwablah Kwadzokpui, Felicia Eyram Adrah, Hanyabui Bless, Latif Mohammed, Ahmed Tijani Bawah, Huseini Wiisibie Alidu

**Affiliations:** ^1^Department of Medical Laboratory Sciences, School of Allied Health Sciences, University of Health and Allied Sciences, Ho-, Ghana; ^2^Clinical Chemistry Unit, Ho Teaching Hospital Laboratory, Ho-, Ghana

## Abstract

**Methods:**

A cross-sectional study was conducted among 185 participants: 88 premenopausal and 97 postmenopausal women obtaining healthcare service from Ho Teaching Hospital (HTH) and Ho Municipal Hospital from November 2018 to January 2020. Questionnaires were administered, and direct anthropometric measurements were taken. Blood samples were collected between 8:00 am and 10:00 am after overnight fast (12 to 18 hours; ≥8 hours) to assess fasting blood glucose, fasting lipids, alanine aminotransferase (ALT), aspartate aminotransferase (AST), and gamma-glutamyl transferase (GGT) concentrations at HTH laboratory using standard measuring procedures. This study in diagnosing metabolic syndrome and nonalcoholic fatty liver disease employed the National Cholesterol Education Program Adult Treatment Panel III (NCEP-ATPIII) criteria and the Bedogni fatty liver index algorithm, respectively.

**Results:**

The overall prevalence of MetS and NAFLD was 24.86% and 40.00% using NCEP-ATPIII and Bedogni fatty liver index algorithm, respectively. The prevalence of MetS and NAFLD among postmenopausal women was 32.99% and 49.48%, respectively, higher than 15.91% and 29.55%, respectively, observed among premenopausal women. The most prevalent MetS component among the study population was abdominal obesity (68.65%) which was significantly higher among the postmenopausal women (82.47%) than premenopausal women (53.41%) (<0.001). Hyperglycemia and hypertension were the major significant risk factors for developing MetS among premenopausal women whereas high triglyceride was the highest risk factor found among the postmenopausal women. Obesity and abdominal obesity were the most likely risk factors for developing nonalcoholic fatty liver disease among both premenopausal and postmenopausal women. Comorbidities of MetS and NAFLD were significant risk factors for developing cardiovascular diseases (CVD) (OR = 5.2, 95%CI = 2.2-12.4; *p* < 0.001).

**Conclusion:**

This study established a significant association between coronary artery disease and comorbidities of MetS and NAFLD among the studied participants. Both conditions were found to be more prevalent among postmenopausal women compared to premenopausal women. Abdominal obesity was the most prevalent MetS component among the population. Women should be monitored for the two conditions and be educated on adopting healthy lifestyles to minimize the incidence of these conditions.

## 1. Introduction

Metabolic syndrome (MetS) is a subject that has received a lot of interest, due to the increasing association with cardiovascular morbidity and mortality [1]. The syndrome is defined as a cluster of key risk factors vis-à-vis abdominal obesity, dyslipidemia, hyperglycemia, and hypertension in a single individual [2]. The condition described as Insulin Resistance Syndrome, Deadly Quartet, or Syndrome X [3] has been reported to be caused by insulin resistance or obesity [4]. The incidence depends on lifestyle, genetic composition of the people, and the criteria for defining the syndrome (NCEP-ATPIII, WHO, and IDF) [5]. The prevalence of the condition has been identified to be high in developed nations because of increased physical inactivity and eating energy-laden foods, which leads to high rates of obesity [1]. The global prevalence is between 7.9% and 43.0% in males and 7.0% and 56% in females [6]. In Africans, the prevalence ranges from 0% to about 50.0% or even higher depending on the population [1]. In Ghana, the prevalence of hypertension [3], diabetes mellitus (DM) [5], hyperlipidemia, and obesity [7] which are individual components of MetS are on the increase [3]. A study conducted among CVD patients in Accra, Ghana, reported that the syndrome is significantly high in women (69.0%) than men (40.7%) [3]. Fatty liver disease (FLD) is a condition in which fat is accumulated in the liver [8]. There are two main types: alcoholic and nonalcoholic fatty liver disease (NAFLD) [9]. NAFLD is regarded as a common and fast-growing liver disease globally with an increase in both mortality and morbidity [10]. The estimated prevalence of NAFLD is thought to be around 25.0-35.0% across the globe and affects largely different proportions of men and women [10]. The prevalence and severity of NAFLD has been documented to be influenced by factors such as diabetes, obesity, age, and gender [11], and the proportion is generally higher in men than in women but however increases in women than men during menopause [11]. Patients with type 2 diabetes mellitus (T2DM) are at high risk of developing NAFLD progressing to advanced liver diseases, such as fibrosis, cirrhosis, and hepatocellular carcinoma [9]. Metabolic syndrome is an important risk factor for heart disease and has threefold increased risk of coronary heart disease, stroke, and increased cardiovascular mortality [5]. In Ghana, there is paucity of published studies on the prevalence of metabolic syndrome and nonalcoholic fatty liver disease among pre- and postmenopausal women. Due to the high risk of mortality from MetS [12, 13] and NAFLD [14], this study was conducted to determine the prevalence, risk factors, and risks of comorbidities of the MetS and NAFLD on cardiovascular health among premenopausal and postmenopausal women in Ho Municipality.

## 2. Materials and Methods

### 2.1. Study Design/Eligibility Criteria

A cross-sectional study was conducted at the Out Patient Department (OPD) of Ho Teaching Hospital (HTH) and Ho Municipal Hospital in Ho, Ghana, from November 2018 to January 2019 to determine the prevalence of MetS and NAFLD among pre- and postmenopausal women who attended these hospitals. A total of 185 (using G^∗^Power 3.1.9.4 statistical software, two tailed, 0.5 effect size, 0.05 alpha error probability, and 0.8 power [15]) conveniently selected women comprising 88 premenopausal and 97 postmenopausal women were recruited for this study upon passing the required inclusion. Women who were menstruating regularly, i.e., have their menses every month, were considered premenopausal women while postmenopausal women were women who had more than 12 months of amenorrhea. Pregnant women, women who experienced amenorrhea for 12 months since last menstruation (menopause), and women on admission were excluded from the study. Women with clinical history of disorders of the ovaries, pituitary gland, hypothalamus, or uterus were excluded from the study. Participants with inconclusive FLI scores (FLI score between 30 and 60) were also excluded from the study and replaced. Only two of such result was recorded and therefore excluded from the overall analysis. Participants who consumed alcohol significantly, thus consuming more than 40 g/day of alcohol for women (equivalent to 4 bottles of beer), were excluded from the study [16]. Participation of women was voluntary. Informed consent was obtained from each of the women after a thorough explanation of the study was done in a language (Ewe, Twi, or English) they understood. Only premenopausal and postmenopausal women receiving healthcare service at Ho Teaching Hospital and Ho Municipal Hospital and who were residents of Ho Municipality were included in this study.

### 2.2. Data Collection

A semi-structured questionnaire was administered to the patients in the language they understood to ascertain their sociodemographic details. Upon arriving at the hospital, patients were provided a comfortable seat to sit down and to relax for about 39 minutes after having to walk for a distance to the facility; this was to eliminate compromised blood pressure outcomes as a result of increased heart rate due to walking. A fully automated blood pressure monitor (Omron Automated Blood Pressure Monitor, HEM-71217, Japan) was used to measure the blood pressure (BP) of each participant twice on the left arm supported at heart level, and mean diastolic and systolic blood pressures were determined. A multipurpose weight and height scale (Yongkang Zhezhong Weighing Apparatus, China) was used to measure body weight of the participants to the nearest 0.1 kg and height to the nearest 0.1 cm, with participants standing erect, back straight, heels together, barefooted, and in light weighted clothing. Body mass index (BMI) was calculated as weight (kg) divided by height squared (m^2^). Waist circumference (WC) (cm) was assessed at the end of expiration, with a Gulick II spring-loaded measuring tape (Gay Mills, WI) midway between the inferior angle of the ribs and the suprailiac crest just below the level of the umbilicus [7]. BMI (kg m^−2^) was categorized according to Centers for Disease Control and Prevention (CDC), underweight (BMI < 18.5), normal weight (BMI = 18.5-24.9), overweight (BMI = 25.0-29.9), and obese (BMI ≥ 30) [17]. Five (5) mL of the venous blood sample was collected from the participant's median cubital vein after overnight fast (12-16 hours) between 8 am to 10 am. Four (4) mL was dispensed into a serum separator tube and 1 mL into a fluoride oxalate tube. Serum and plasma were obtained by centrifuging the samples at 2500 rpm for 5 minutes, pipetted into cryotubes, and stored at -20°C until analysis. Gamma-glutamyl transferase (GGT), fasting plasma glucose (FPG), total cholesterol (TC), triglyceride (TG), high-density lipoprotein cholesterol (HDL-C), alanine aminotransferase (ALT), and aspartate aminotransferase (AST) concentrations were estimated using a Selectra ProS chemistry analyzer, adhering to the reagent manufacturer's instructions (ELITech Clinical Systems). Very low-density lipoprotein cholesterol (VLDL-C) and low-density lipoprotein cholesterol (LDL-C) were calculated using the Frederickson-Friedewald formula [18]: LDL‐C = TC–HDL‐C–VLDL‐C, where VLDL‐C = TG/2.2 and units of measurements were in mmol/L.

### 2.3. Metabolic Syndrome Diagnostic Criteria and Fatty Liver Index (FLI) Calculation

MetS was defined in this study according to National Cholesterol Education Program Adult Treatment Panel III (NCEP-ATPIII) criteria where MetS was present in an individual if three out of the five parameters below are present in the person: abdominal obesity (waist circumference > 88 cm for women), high concentration of TG ≥ 1.7 mmol/L, low HDL-C (<1.0 mmol/L for women), high BP (systolic BP ≥ 130 mmHg or diastolic BP ≥ 85 mmHg or treatment of hypertension), and increased FPG ≥ 6.1 mmol/L.[19]

The fatty liver index was calculated based on the fatty liver index (FLI) algorithm by Bedogni and colleagues [20] where BMI, waist circumference, TG, and GGT were entered into an online calculator to estimate the fatty liver index:

(1) $$\begin{array}{c} \text{FLI}=\frac{\left(e^{0.953}\times \text{loge}\left(\text{TG}\right)+0.139\times \text{BMI}+0.718\times \text{loge}\left(\text{GGT}\right)+0.053\times \text{WC}-15.745\right)}{\left(1+e^{0.953}\times \text{loge}\left(\text{TG}\right)+0.139\times \text{BMI}+0.718\times \text{loge}\left(\text{GGT}\right)+0.053\times \text{WC}-15.745\right)\times 100}. \end{array}$$

Triglyceride measurement was done in mmol/L, GGT in U/L, BMI in kg/m^2^, and waist circumference in cm. The FLI score ranges from 0 to 100. FLI < 30 rules out fatty liver, and FLI ≥ 60 rules in fatty liver; however, FLI between 30 and 60 was interpreted as inconclusive and therefore not included in this study [20].

### 2.4. Cardiovascular Risk Calculation

The coronary risk was calculated by dividing total cholesterol over high-density lipoprotein multiplied by 1.3808 (TC/HDL × 1.3808) [21].

### 2.5. Statistical Analysis

Unique codes were assigned to each participant in order to ensure data security and confidentiality. Normality of all continuous variables was tested. Continuous parametric variables were expressed as their mean ± standard deviation; continuous nonparametric variables were expressed as median (minimum and maximum) while categorical variables were expressed as frequencies and percentages. Comparisons of parameters were performed using the unpaired *t*-tests, Mann–Whitney *U* test, chi-squared (*χ*2) test, or Fisher's exact test where appropriate. A *p* < 0.05 was considered statistically significant for all analyses. Data were analyzed using the SPSS statistical software version 22.00, GraphPad Prism statistical software version 6, and Microsoft Excel 2016.

### 2.6. Ethical Issues

Ethical approval for the study was obtained from the Research Ethics Committee of the University of Health and Allied Sciences with Ethical Clearance Certificate Number UHAS-REC A.4 [226] 18-19 and the authorities in the two facilities: Ho Teaching Hospital and Ho Municipal Hospital. The study was explained in the language of the patients who were well oriented, and written informed consent was obtained. Patients were assigned study identification numbers (IDs) which were used throughout the study. The IDs were generated by using the initials of the hospital under investigation and chronological number according to how the patients were recruited. Only the researcher and supervisors had access to study data.

## 3. Results and Analysis

### 3.1. Sociodemographic Data Characteristics of Study Participants

One hundred and eighty-five participants with a mean age of 47.67 ± 16.18 years were recruited in this study. Out of the 185 participants, 88 and 97 were premenopausal and postmenopausal women, respectively. Most of the participants' ages fell between 46 and 65 years. Forty-two percent (77/185) had attained at least secondary education at the time of the study. A greater proportion (55.1%) of the participants worked in the informal sector, and the majority of them reported to be single (99 (53.5%)). Among the premenopausal women, 40 (45.45%) fell within the age category of 30-45 years while the postmenopausal women were within 46-65 years of age. The differences in the proportions of the age groups among the pre- and postmenopausal women were statistically significant (*p* < 0.001). A higher percentage (34.09%) of the premenopausal women attained a higher level of education (tertiary) compared to (16.49%) postmenopausal women. Generally, employment status was leveled between the two studied groups (Table 1).

### 3.2. Anthropometric, Hemodynamic, and Biochemical Parameters among the Participants

Except for weight, HDL-C and AST, all the anthropometric data, and hemodynamic and biochemical parameters showed statistically significant differences between pre- and postmenopausal women. In general, postmenopausal women presented with higher anthropometric, hemodynamic, and biochemical outcomes except height which was significantly higher among the premenopausal group compared to the postmenopausal group (Table 2).

### 3.3. Prevalence of the Components of Metabolic Syndrome among the Study Participants

In this study, abdominal obesity among the study participants had the highest prevalence (68.65%) followed by hyperglycemia (34.59%), high blood pressure (27.03%), low high-density lipoprotein (21.62%), and elevated triglyceride (15.68%). All the components were observed to be more prevalent in postmenopausal women except low HDL, which was more prevalent in premenopausal women. The increased prevalence of abdominal obesity, HBP, and hyperglycemia observed among the postmenopausal women was statistically significant (Table 3).

### 3.4. Prevalence of Metabolic Syndrome and Fatty Liver Disease among Premenopausal and Postmenopausal Women in Ho Municipality

As shown in Table 4, out of the total population studied, 46 (24.86%) were diagnosed with metabolic syndrome with the premenopausal women recording 15.91% and the postmenopausal women recording 32.99% prevalence. The prevalence of nonalcoholic fatty liver disease among the total participants was 40.00%. However, the prevalence of 29.55% and 49.48% was determined among the premenopausal and postmenopausal women, respectively. In this study, participants' menopausal status was significantly associated with both MetS (*p* = 0.010) and NAFLD (*p* = 0.007).

### 3.5. Risk Factors of Metabolic Syndrome among Pre- and Postmenopausal Women in Ho Municipality

This study showed BMI, WC, FBG, BP, age, TG, HDL-C, and alcohol consumption to be significantly associated with MetS among the premenopausal women. In postmenopausal women, MetS was associated with participants' BMI, WC, FPG, BP, TG, and HDL. The odds of MetS among obese premenopausal women was found to be 7.6 times higher compared to that of a normal premenopausal individual (95%CI = 1.5-38.8). This observation, however, differs slightly in the case of postmenopausal women where an obese woman is only about four times (OR = 4.1, 95%CI = 1.0-16.8) more likely to develop MetS. In addition, the odds of developing MetS in both pre- and postmenopausal women who had abdominal obesity, high fasting plasma sugar, high TG, and low HDL-C and who were hypertensive was significantly (statistical significance indicated with star) high compared to that of their counterparts within normal blood pressure (Table 5).

### 3.6. Risk Factors of Nonalcoholic Fatty Liver Disease among Pre- and Postmenopausal Women in Ho Municipality

In this study, a statistically significant association between NAFLD and BMI, WC, and age was established among the premenopausal women while BMI, WC, TG, and BP were significantly associated with NAFLD in postmenopausal women. The likelihood of obese premenopausal women developing NAFLD was significantly high (OR = 17.4, 95%CI = 4.3-71.1) but even higher among obese postmenopausal women (OR = 180.0, 95%CI = 17.3-1872.3) when compared to participants with normal status. The odds of premenopausal women who fell within the age category of 30-45 years developing NAFLD was 7 times higher (OR = 7.1, 95%CI = 1.9-27.4) than those less than 30 years of age while those aged 46-65 years were 5.8 times more likely to develop NAFLD (OR = 5.8, 95%CI = 1.2-27.6). The odds of developing NAFLD by women who had abdominal obesity according to the findings from this study is 45.5 times higher (OR = 45.5, 95%CI = 5.8-358.6) among premenopausal women when compared to the normal and 25 times higher among the postmenopausal women when compared to the normal high (OR = 25.0, 95%CI = 3.2-197.1). High TG concentrations render postmenopausal women 7.7 times more likely of developing NAFLD compared to the similar populations with normal TG levels. Though abdominal obesity was statistically associated with NAFLD among both premenopausal women and postmenopausal women, the odds of developing FLD in the premenopausal women was not statistically significant (Table 6).

### 3.7. Risk Associated with Comorbidities of MetS and NAFLD on Cardiovascular Health in Premenopausal and Postmenopausal Women in Ho Municipality

In this study, 18.38% of the studied participants were diagnosed with both MetS and NAFLD. Coronary risk was higher among 38.24% of participants with comorbidities of MetS and NAFLD than 10.60% of participants with a single condition. The association between coronary risk and interaction between metabolic syndrome and NAFLD was highly significant (*p* value < 0.001). The likelihood of developing coronary risk in the presence of both conditions was 5.2 times (OR = 5.2, 95%CI = 2.2-12.4; *p* value < 0.001) higher that than in the presence of a single condition. Coronary risk was significantly associated with comorbidities of MetS and NAFLD in this study (Table 7).

## 4. Discussion

Currently, CVD has become a major cause of mortality of women in the world and it has been shown that being affected by MetS increases the risk of CVD [22]. Nonalcoholic fatty liver disease (NAFLD) which has been recognized as a hepatic manifestation of metabolic syndrome linked with insulin resistance is a common cause of chronic liver disease worldwide and is rapidly becoming a major public health problem [23]. The overall prevalence of MetS estimated in this study population was 24.86% using NCEP-ATPIII criteria. This finding, as indicated in Table 4, is in agreement with Arthur et al. (2013), who reported a prevalence of 25% among pre- and postmenopausal women in Kumasi, Ghana [7]. A lower prevalence of 15.0% (NCEP-ATPIII) among the rural population in Ghana and 12.7% in the Nigerian population has been documented [24]. Meanwhile, a rather higher prevalence of 35.9% was recorded in Tunisia among a similar population [22]. Possible explanation to these dissimilarities in prevalence rates could be sociocultural practices, lifestyle habits, and genetic variations. The varying prevalence among women largely depends on the characteristics of the population and the diagnostic criteria applied [7], which has been reported to vary from country to country [22]. In this study, the prevalence of MetS was higher among the postmenopausal group (32.99%) compared to their premenopausal counterparts (15.91%), a comparable finding among a similar population in Kumasi, Ghana [7]. Elsewhere, Tunisia reported a prevalence of 45.7% and 25.6% whereas Brazil estimated a prevalence of 22.2% and 9.4% among pre- and postmenopausal women, respectively [22, 25]. Our finding is consistent with a study conducted by Arthur et al. [7]. Possible elucidation for the outcome from this study could be due to the fact that postmenopausal women have a decreased estrogen hormone known to cause buildup of visceral fat [22] which produces inflammatory cytokines and free fatty acids that drain directly into the liver through the portal circulation [5] resulting in the overproduction of very low-density lipoproteins, predisposing women to atherogenic dyslipidemia (elevated triglyceride and low HDL-cholesterol level) [7]. In addition, the disparities observed in the prevalence rates could be ascribed to factors such as sociocultural practices, lifestyle habits, geographic or climatic differences, and genetic compositions of the participants. The prevalence of FLD among the total population of this study was 40.0% (Table 4). This prevalence conforms to the prevalence estimated (43.9%) among the Chinese population [26]. North America recorded a higher 47.2% prevalence [27] while Korea estimated a rather lower prevalence of 20.7% [28] among related population. The variation in this study compared to other works may be due to the diverse diagnostic methods employed vis-à-vis ultrasound which could reliably diagnose NAFLD when used with appropriate clinical risk factors and a greater than 33% steatosis of the liver and liver biopsy which is considered the gold standard with an added advantage of staging the degree of hepatic fibrosis [29]. It could also be attributed to different ethnicities and cultural practices. In about a decade ago, the prevalence of NAFLD among women was reported alarming [27], a situation that seems not too different from the findings from this study. In this study, a significantly higher prevalence of NAFLD (*p* = 0.007) was recorded in postmenopausal women (49.48%) compared to their premenopausal counterparts (29.55%). This is in agreement with previous studies which indicated that the prevalence of NAFLD in postmenopausal women was higher than that in premenopausal women [27, 28, 30]. A possible underlying factor for this finding may be due to estrogen hormones known to be accountable for the preferential fat accumulation in the hips and thigh region as well as the elevation in central fat accumulation despite its phenomenal decline during the menopausal transition period [30]. A significantly high (*p* < 0.001) association between coronary risk and comorbidity of metabolic syndrome and nonalcoholic fatty liver disease was found in this study. Participants who had both MetS and NAFLD were five times at risk of developing cardiovascular risk (OR = 5.2, 95%CI = 2.2-12.4, *p* < 0.001) compared to those who had only one of the condition or none of the conditions (Table 7). A strong association between MetS and CVD has been reported in Accra, Ghana [3], as well as in North Africa and South America where NAFLD and MetS were shown to be associated with increased risk of cardiovascular disease [31, 32]. This study also indicated that hyperglycemia and hypertension were the major risk factors significantly associated with MetS among the premenopausal women (OR = 20.4, 95%CL = 5.2-80.3; *p* value < 0.001). In contrast, postmenopausal women with elevated TG were about 21 times likely of developing MetS (OR = 20.7, 95%CI = 5.4-79.7; *p* value < 0.001). Compared to the normal, premenopausal women with abdominal obesity were 15 times likely to develop MetS (OR = 15.3, 95%CI = 1.9-123.0) but 45 times likely to develop NAFLD (OR = 45.5, 95%CI = 5.8-358.6). Postmenopausal women with abdominal obesity on the other hand were about 25 times at risk of developing NAFLD (OR = 25.0, 95%CI = 3.2-197.1) and 180 times at risk of developing NAFLD if obese (OR = 180.0, 95%CI = 17.3-1872.3). The increased abdominal obesity-related NAFLD risk found in this study compares well with that reported among pre- and postmenopausal Korean women where abdominal obesity, elevated triglyceride, and hyperglycemia were found to be risk factors of nonalcoholic fatty liver disease [28].

## 5. Conclusion

MetS and NAFLD are fast becoming a public health concern. In this study, MetS and NAFLD were found to be more prevalent among postmenopausal women compared to premenopausal women. Abdominal obesity was the most prevalent component among the population. The interaction between the two conditions (MetS and NAFLD) was found to be significantly associated with coronary risk among the studied participants.

## Figures and Tables

**Table 1:** Sociodemographic features stratified by pre- and postmenopausal status.

	Total *N* (%)	Premenopausal *n* (%)	Postmenopausal *n* (%)	*p* value
	185 (100.00)	88 (47.57)	97 (52.43)	
Age group				
<30	32 (17.30)	32 (36.36)	0 (0.00)	<0.001
30-45	48 (25.95)	40 (45.45)	8 (8.25)	
46-65	82 (44.32)	16 (18.18)	66 (68.04)	
>65	23 (12.43)	0 (0.00)	23 (23.71)	
Educational status				
None	17 (9.19)	4 (4.55)	13 (13.40)	0.002
Basic	45 (24.32)	26 (29.55)	19 (19.59)	
Secondary	77 (41.62)	28 (31.82)	49 (50.52)	
Tertiary	46 (24.86)	30 (34.09)	16 (16.49)	
Occupation				
Unemployed	60 (32.43)	27 (30.68)	33 (34.02)	0.635
Informal sector	102 (55.14)	48 (54.55)	54 (55.67)	
Formal sector	23 (12.43)	13 (14.77)	10 (10.31)	
Marital status				
Single	99 (53.51)	49 (55.68)	50 (51.55)	0.658
Married	86 (46.49)	39 (44.32)	47 (48.45)	

Data is presented as frequencies and percentages in parentheses. *p* value is significant at <0.05.

**Table 2:** Anthropometric, hemodynamic, and biochemical factors among study participants.

Parameter	Total	Postmenopausal	Premenopausal	*p* value
Anthropometric				
Weight (kg)	69.00 (39.00-150.00)	70.00 (39.00-121)	69.50 (47.00-106.00)	0.377
Height (m)	1.58 (1.44-1.85)	1.56 (1.44-1.85)	1.61 (1.44-1.83)	0.003
WC (cm)	96.00 (54.00-138.00)	100.00 (54.00-138.00)	89.50 (57.00-138.00)	<0.001
BMI (kg/m^2^)	27.10 (14.30-47.00)	28.50 (14.30-47.40)	25.70 (19.50-43.20)	0.028
Hemodynamic				
SBP (mmHg)	127.10 (90.00-207.00)	131.00 (94.00-188.00)	118.0 (90.0-207.0)	<0.001
DBP (mmHg)	80.00 (51.00-127.00)	82.00 (60.00-115.00)	78.0 (51.0-127.0)	0.016
Biochemical				
FBG (mmol/L)	5.32 (3.29-22.20)	6.24 (3.69-17.14)	4.87 (3.48-16.28)	0.003
TC (mmol/L)	5.50 (2.47-10.50)	5.63 (3.20-10.50)	5.23 (2.47-8.79)	0.004
TG (mmol/L)	0.99 (0.14-3.54)	1.17 (0.14-3.54)	0.76 (0.37-2.42)	<0.001
HDL (mmol/L)	1.24 ± 0.35	1.31 ± 0.35	1.24 ± 0.35	0.092
GGT (U/L)	25.00 (4.00-148.00)	29.00 (4.00-148.00)	20.00 (7.00-118.00)	0.001
AST (U/L)	21.00 (10.00-95.00)	21.00 (12.00-95.00	22.00 (10.00-67.00)	0.191
ALT (U/L)	15.00 (4.00-67.00)	16.00 (6.00-67.00)	13.00 (4.00-150.00)	0.008
VLDL (mmol/L)	0.45 (0.06-1.61)	0.53 (0.06-1.61)	0.35 (0.17-1.10)	<0.001
LDL (mmol/L)	3.75 (1.30-8.15)	3.91 (3.20-10.50)	3.64 (1.48-6.37)	0.007

Data is presented as median with minimum and maximum in parentheses for nonparametric data and mean ± standard deviation for parametric data. *p* value is significant at <0.05. BMI: body mass index; WC: waist circumference; SBP: systolic blood pressure; DBP: diastolic blood pressure; FBG: fasting blood glucose; HDL: high-density lipoprotein; LDL: low-density lipoprotein; VLDL: very low-density lipoprotein; AST: aspartate aminotransferase; ALT: alanine aminotransferase.

**Table 3:** Prevalence of components of metabolic syndrome among the study participants.

Components	Total *N* (%)	Postmenopausal *n* (%)	Premenopausal *n* (%)	*p* value
	185 (100.00)	97 (52.43)	88 (47.57)	
Abdominal obesity	127 (68.65)	80 (82.47)	47 (53.41)	<0.001
High blood pressure	50 (27.03)	35 (36.08)	15 (17.05)	0.005
Hyperglycemia	64 (34.59)	49 (50.52)	15 (17.05)	<0.001
Elevated TG	29 (15.68)	19 (19.59)	10 (11.36)	0.157
Low HDL	40 (21.62)	17 (17.53)	23 (26.14)	0.210

Data is presented as frequencies and percentages in parentheses. *p* value is significant at <0.05.

**Table 4:** Prevalence of MetS and NAFLD among pre- and postmenopausal women.

Menopause status	Total	MetS	Non-MetS	*p* value

Pre- & postmenopausal	185 (100.00)	46 (24.86)	139 (75.14)	
Premenopausal	88 (47.57)	14 (15.91)	74 (84.09)	0.010
Postmenopausal	97 (52.43)	32 (32.99)	65 (67.01)	
	Total	NAFLD	Non-NAFLD	
Pre- & postmenopausal	185 (100.00)	74 (40.00)	111 (60.00)	
Premenopausal	88 (47.57)	26 (29.55)	62 (70.45)	0.007
Postmenopausal	97 (52.43)	48 (49.48)	49 (50.52)	

Data are presented as frequencies and percentages. *p* value is significant at <0.05. Fisher's Exact test was used to compute the *p* value.

**Table 5:** Risk factors of metabolic syndrome among pre- and postmenopausal women.

Parameters	Premenopausal women			Postmenopausal women		
	MetS	OR (95% CI)	*p* value	MetS	OR (95% CI)	*p* value
Total	14 (15.91)			32 (32.99)		
BMI						
Obese	9 (64.29)	7.6 (1.5-38.8)	0.032	19 (59.38)	4.1 (1.0-16.8)	0.047
Overweight	3 (21.43)	2.4 (0.4-15.6)		10 (31.25)	1.4 (0.3-6.1)	
Normal	2 (14.29)	1		3 (9.38)	1	
Underweight	0 (0.00)	1.6 (0.1-47.0)		0 (0.00)	0.8 (0.0-20.0)	
WC						
AO	13 (92.86)	15.3 (1.9-123.0)^∗^	0.001	31 (96.88)	10.1 (1.3-80.2)^∗^	0.009
Normal	1 (7.14)	1		1 (3.13)	1	
FPG						
High	9 (64.29)	20.4 (5.2-80.3)^∗^	<0.001	27 (84.38)	10.6 (3.6-31.2)^∗^	<0.001
Normal	5 (35.71)	1		5 (15.63)	1	
BP						
High	9 (64.29)	20.4 (5.2-80.3)^∗^	<0.001	22 (68.75)	8.8 (3.4-23.1)^∗^	<0.001
Normal	5 (35.71)	1		10 (31.25)	1	
Age						
<30	0 (0.00)	0.0 (0.0-0.8)^∗^	0.009	—	—	
30-45	10 (71.43)	1		4 (12.50)	2.0 (0.5-8.8)	0.462
46-65	4 (28.57)	1.0 (0.3-3.8)		22 (68.75)	1	
>65				6 (18.75)	0.7 (0.2-2.0)	
TG						
High	5 (35.71)	7.7 (1.9-31.7)^∗^	0.008	16 (50.00)	20.7 (5.4-79.7)^∗^	
Normal	9 (64.29)	1		16 (50.00)	1	<0.001
HDL						
Low	10 (71.43)	11.7 (3.2-43.3)^∗^	0.001	14 (43.75)	16.3 (4.2-63.2)^∗^	
Normal	4 (28.57)	1		18 (56.25)	1	<0.001
Alcohol intake						
Ever drunk	9 (64.29)	5.2 (1.6-17.5)^∗^	0.010	10 (31.25)	0.8 (0.3-1.9)	0.655
Never drink	5 (35.71)	1		22 (68.75)	1	

Data are presented as frequencies and percentages in parentheses. *p* value is significant at <0.05. OR = odds ratio; 95% CI = 95% confidence interval; BMI = body mass index; WC = waist circumference; AO = abdominal obesity; FPG = fasting plasma glucose; BP = blood pressure; TG = triglyceride; HDL = high-density lipoprotein, ^∗^Significant odds ratio, *p* value < 0.05.

**Table 6:** Risk factors of fatty liver disease among pre- and postmenopausal women.

Parameters	Premenopausal women			Postmenopausal women		
	FLD	OR (95% CI)	*p* value	FLD	OR (95% CI)	*p* value
Total	26 (29.55)			48 (49.48)		
BMI						
Obese	22 (84.62)	17.4 (4.3-71.1)^∗^	<0.001	36 (75.00)	180.0 (17.3-1872.3)^∗^	<0.001
Overweight	4 (15.38)	15.9 (0.8-311.5)		11 (22.92)	5.7 (0.7-48.4)	
Normal	0 (0.00)	1		1 (2.08)	1	
Underweight	0 (0.00)	0.6 (0.0-14.2)		0 (0.00)	2.1 (0.064-66.3)	
WC						
AO	25 (96.15)	45.5 (5.8-358.6)	<0.001	47 (100.00)	25.0 (3.2-197.1)^∗^	<0.001
Normal	1 (3.85)	1		1 (0.00)	1	
FBG						
Hyperglycemia	7 (26.92)	2.5 (0.8-7.9)	0.128	29 (60.42)	2.2 (1.0-5.0)	0.054
Normal	19 (73.08)	1		19 (39.58)	1	
TG						
High	6 (23.08)	4.4 (1.1-17.0)	0.059	16 (33.33)	7.7 (2.1-28.5)^∗^	0.001
Normal	20 (76.92)	1		32 (66.67)	1	
HDL						
Low	10 (38.46)	2.4 (0.9-6.4)	0.113	11 (22.92)	2.1 (1.0-6.3)	0.167
Normal	16 (61.54)	1		37 (77.08)	1	
BP						
High	7 (26.92)	2.5 (0.8-7.8)	0.128	23 (47.92)	2.8 (1.2-6.7)^∗^	0.016
Normal	19 (73.08)	1		25 (52.08)	1	
Age						
<30	3 (11.54)	1	0.007			
30-45	17 (65.38)	7.1 (1.9-27.4)		2 (4.17)	1	0.351
46-65	6 (23.08)	5.8 (1.2-27.6)		34 (70.83)	3.2 (0.6-17.0)	
>65	—	—		12 (25.00)	2.3 (0.5-19.7)	

Data are presented as frequencies and percentages in parentheses. *p* value is significant at <0.05. OR = odds ratio; 95% CI = 95% confidence interval; BMI = body mass index; WC = waist circumference; AO = abdominal obesity; FBG = fasting blood glucose; BP = blood pressure; TG = triglyceride; HDL = high-density lipoprotein. ^∗^Significant odds ratio, *p* value < 0.05.

**Table 7:** Coronary risk and interaction between metabolic syndrome and fatty liver disease.

Interactions	Total	Coronary risk		OR (95% CI)	*p* value
		High	Normal		
Mets and NAFLD	34 (18.38)	13 (38.24)	21 (61.76)	5.2 (2.2-12.4)^∗^	<0.001
Single conditions	151 (81.62)	16 (10.60)	135 (89.40)	1	
Total	185 (100.00)	29 (15.68)	156 (84.32)		

Data is presented as frequencies and percentages in parentheses. *p* value is significant at <0.05. ^∗^Significant odds ratio.

## Data Availability

Data is available on reasonable request.

## Additional Points

*Limitations*. The results of NAFLD were not based on biopsy, the proven goal standard diagnostic method. *Recommendation*. Women should be educated on the risk factors of MetS and NAFLD to reduce the occurrence of the conditions. Further studies should be undertaken, utilizing liver biopsy in diagnosing as well as staging liver fibrosis in NAFLD individuals.
